# The Small-Pox Panic

**Published:** 1902-02-15

**Authors:** 


### The Small-pox Panic.

So free has London been from small-pox for a considerable number of years, that the revival of the disease which has taken place during the past few months is, from a public health point of view, a cause for much disappointment, but there is not the slightest excuse for the suggestion that London is in the throes of such an epidemic as to be a place dangerous to visit. Yet such is the notion which is gradually being spread abroad, largely in consequence, as we cannot but think, of the prominence given to the topic by the daily papers. By devoting a large amount of space every morning to an elaborate display of the small-pox returns, by detailing a crowd of details concerning the precautionary measures taken by the various sanitary authorities, and by giving admission to endless letters on the subject, an artificial and factitious interest and excitement about small-pox is being aroused which is most injurious, for the matter has now got to such a pitch that people outside London are panic stricken and are afraid either to come to London themselves or to allow members of their families to do so. People who wish to come to London are asking "Is it safe?" People going south ask "Will there be any risk in going through London?" and even men of business are becoming nervous and will not stop a night in London if they can help it. This is a most serious matter. London has before it the possibility and the hope of a brilliant season, and if all goes well we shall receive visitors from every quarter of the globe. London trade may thus receive a great and much needed impetus. But all is likely to be wrecked by this notion of an epidemic which has its origin, not, as we think, in the extent of the outbreak, but in the prominence given to it by the newspapers.

The last occasion on which London suffered from any considerable epidemic of small-pox was in 1871, during which year 7,912 persons died from the disease. The present outbreak has lasted close on six months, and so far the deaths have amounted to 524. During the 1871 epidemic the death-rate was only slightly above the ordinary small-pox mortality which prevailed almost continuously in times gone by, before vaccination was introduced. This brings us round to what really is the bottom of the mischief, namely, that if our good friends the editors instead of exciting the popular imagination about small pox would with one accord put their shoulders to the wheel and urge vaccination and re-vaccination they would do some real good and might between them save London its "season."

Some years ago Gloucester got in a "tight corner" about small-pox, nearly one-twentieth of its inhabitants having suffered from the disease. Think of what one in twenty would mean if applied to London. Think of 200,000 cases of small-pox! and compare that with the statement made by Mr. Scovell at the meeting of the Asylums Board last Saturday, that since August 10th the total number admitted was 3,001. Well, Gloucester was in a tight corner, and what its inhabitants did was to organise a house-to-house visitation and to vaccinate right and left. Out of a population of 42,000, upwards of 36,000 vaccinations were performed. The bottom was knocked out of the epidemic, and in a short time Gloucester was happy once again. So far as the morbid fear of small-pox, and the exaggerated views which now prevail as to the danger of visiting London are concerned, we may point out that we are infinitely better prepared to meet an epidemic than we were in 1871, when only about a third of all the cases could be isolated, and about 4,000 cases, capable of communicating the disease, were at large in London. The Medical Officer of Health for London—a well-known sanitary authority of much experience—stated the other day that if all those who are susceptible to small-pox would be at once vaccinated, small-pox would be stamped out in three weeks' time. Small-pox is the very type of a preventable disease. Why, then, to use the words of the King, is it not prevented? And we may add, why does not the nation at large manifest equal manliness with his Majesty, who has frequently visited the theatres and other places of amusement with members of his family during the last few weeks? If it is safe for the King it must be equally safe for his subjects to reside in the metropolis in existing circumstances.

